# Factors associated with assisted ventilation use in amyotrophic lateral sclerosis: a nationwide population-based study in Korea

**DOI:** 10.1038/s41598-021-98990-x

**Published:** 2021-10-04

**Authors:** Seo Yeon Yoon, Han-Kyoul Kim, Mi Ji Kim, Jee Hyun Suh, Ja-Ho Leigh

**Affiliations:** 1 Department of Physical Medicine and Rehabilitation, Korea University Guro Hospital, Seoul, Republic of Korea; 2 Department of Rehabilitation Medicine, Seoul National University Hospital, Seoul, Republic of Korea; 3 National Traffic Injury Rehabilitation Research Institute, National Traffic Injury Rehabilitation Hospital, Yang-Pyeong, Republic of Korea; 4 Department of Biostatistics and Computing, Yonsei University Graduate School, Seoul, Republic of Korea; 5 Department of Rehabilitation Medicine, College of Medicine, Ewha Womans University, Seoul, Republic of Korea; 6 Department of Rehabilitation Medicine, Seoul National University College of Medicine, 101, Daehak-ro, Jongno-gu, Seoul, 03080 Republic of Korea

**Keywords:** Health care, Neurology

## Abstract

Few studies have investigated the factors associated with assisted ventilation use in amyotrophic lateral sclerosis (ALS) in western countries with a relatively small number of participants. This study aimed to evaluate the factors associated with assisted ventilation use using a large nationwide cohort covering the entire Korean population. We selected patients with primary or secondary diagnoses of ALS (ICD-10 code: G12.21) and a registration code for ALS (V123) in the rare intractable disease registration program. Covariates included in the analyses were age, sex, socioeconomic status and medical condition. Factors associated with non-invasive ventilation (NIV) and tracheostomy invasive ventilation (TIV) were evaluated. Logistic regression analyses were performed using odds ratios and 95% confidence intervals. In total, 3057 patients with ALS were enrolled. During the 6-year follow-up period, 1228 (40%) patients started using assisted ventilation: 956 with NIV and 272 with TIV. There was no significant difference in the assisted ventilation use according to sex, whereas different patterns of discrepancies were noted between the sexes: Females living in non-metropolitan areas showed decreased use of assisted ventilation, whereas high income levels showed a positive relationship with assisted ventilation use only in males. Patients aged ≥ 70 years showed decreased use of NIV. NIV use was more affected by socioeconomic status than TIV, whereas TIV showed a significant relationship with medical conditions such as nasogastric tube insertion and gastrostomy. We found that various factors, including age, socioeconomic status, and medical condition, were related with assisted ventilation use. Understanding the pattern of assisted ventilation use would help set optimal management strategies in patients with ALS.

## Introduction

Amyotrophic lateral sclerosis (ALS) is an adult-onset neurological disorder characterized by the progressive impairment of motor functions due to degeneration of the upper and lower motor neurons. It begins insidiously with focal weakness, especially in the arms or legs, and eventually affects most of the muscles, including the diaphragm. Currently, there is currently no curative treatment for ALS, with respiratory failure being the most common cause of death among patients with ALS^1^. Assisted ventilation is a method wherein machines are used to support a person’s breathing. Over the past few decades, both invasive and non-invasive assisted ventilation methods have been attempted for ALS management, and a consensus has been reached on its beneficial effects on dyspnea, quality of life, and survival among patients with ALS^2–5^.

Although assisted ventilation, especially non-invasive ventilation (NIV), has been considered the treatment of choice in the management of respiratory disturbances in ALS^4,5^, there is no consensus on the ideal indication or timing of its use^4,6^. The American Academy of Neurology guidelines recommend NIV when a patient’s forced vital capacity (FVC) falls below 50% of the predicted value^5^. However, previous studies reported that less than 10% of patients with an FVC below 40% of the predicted value^7^, or only 36% of patients with an FVC below 50%^8^ were using NIV. Several factors have been suggested to be associated with NIV use, including orthopnea, dyspnea, gastrostomy and low body mass index^8,9^. In addition, demographic factors such as sex, age and marital status, were also suggested to affect NIV utilization^10,11^.

There have been a small number of studies that investigated the factors associated with assisted ventilation use in patients with ALS^8,12^. Only few of these studies were published recently^9,12^, and almost all of them were conducted in Western countries^8–11^. Ethnic differences in ALS regarding genetic variation between the European and Korean populations have been reported^13,14^, which could lead to different processes of disease progression and management strategies. On the other hand, socioeconomic costs have been suggested to increase in the management of ALS estimating increased costs due to population aging in the future^15–18^. However, only few reports have assessed assisted ventilation use in terms of socioeconomic status in ALS^8^. Therefore, this study aimed to determine the factors associated with assisted ventilation use in the Korean population using a large nationwide population-based cohort. We evaluated the risk factors for NIV and tracheostomy invasive ventilation (TIV), stratified by sex, with adjustment for socioeconomic status (SES).

## Results

### Baseline characteristics of patients

**Figure 1:**
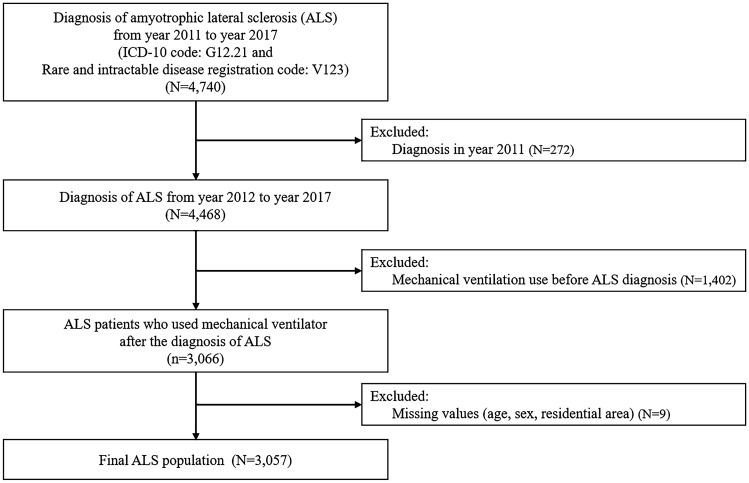
Sample selection flowchart.

A total of 3057 patients with ALS were enrolled in the study (Fig. 1). Among them, 1228 (40%) started assisted ventilation during the 6-year follow-up period, with 956 (31.3%) using NIV and 272 (8.9%) using TIV. Demographic and medical characteristics of patients with ALS according to assisted ventilation use are presented in Table 1. Regarding sex, no significant differences were observed between the two groups. Regarding age, patients aged 60–69 years demonstrated the most frequent use of assisted ventilation, whereas those aged ≥ 70 years showed less use of assisted ventilation. Regarding SES, tertiary hospital, high income and national health insurance (NHI) were related to increased use of assisted ventilation. Regarding medical condition, patients with assisted ventilation had a higher incidence of nasogastric tube insertion, gastrostomy, tracheostomy, and pneumonia than those without assisted ventilation use. The mean survival time of ALS patients with NIV and TIV use was 25.6 ± 18.0 and 26.2 ± 17.3 months, respectively.

**Table 1:** Characteristics of the amyotrophic lateral sclerosis participants with ventilator (N = 1228) and without ventilator (N = 1829).

	No ventilator (N = 1829)		Ventilator (N = 1228)		*p* value
	N	%	N	%	
**Sex**					
Female	745	40.7	494	40.2	0.781
Male	1084	59.3	734	59.8	
**Age group**					
≤ 49	247	13.5	151	12.3	< .001
50–59	437	23.9	352	28.7	
60–69	513	28.0	428	34.9	
≥ 70	632	34.6	297	24.2	
**Regions**					
Capital	347	19.0	271	22.1	0.0012
Metropolitan	390	21.3	306	24.9	
Non-metropolitan	1092	59.7	651	53.0	
**Income level (quartiles)**					
Lowest	509	27.8	241	19.6	< .001
Low-middle	293	16.0	207	16.9	
Middle-high	417	22.8	311	25.3	
Highest	610	33.4	469	38.2	
**Hospital**					
Tertiary Hospital	1234	67.5	969	78.9	< .001
General Hospital	374	20.4	186	15.1	
Others	221	12.1	73	5.9	
**Type of insurance**					
National Health Insurance	1676	91.6	1204	98.0	< .001
Medical aid	153	8.4	24	2.0	
**Nasogastric tube**	279	15.3	379	30.9	< .001
**Gastrostomy**	191	10.2	305	24.8	< .001
**Tracheostomy**	52	2.8	272	22.1	< .001
Pneumonia (N)*	1.009 ± 3.204		2.954 ± 7.352		< .001
CCI score*	1.574 ± 1.480		2.521 ± 1.681		< .001

*Mean ± standard deviation.

### Factors associated with assisted ventilation use in ALS

Table 2 displays the odds ratio (OR) and corresponding 95% confidence intervals (CI) for assisted ventilation obtained from univariate and multivariate logistic regression models. Regarding sex, no significant relationship was found between sex and assisted ventilation use. Regarding age, patients aged ≥ 70 years showed a significant relationship with decreased use of assisted ventilation in all regression models. In the multivariate regression model, gastrostomy (OR = 1.420, 95% CI 1.107–1.822), tracheostomy (OR = 9.310, 95% CI 6.357–13.636), pneumonia (OR = 1.007, 95% CI 1.053–1.102) and CCI score (OR = 1.358, 95% CI 1.288–1.431) were related to an increased risk of assisted ventilation use. Regarding SES, tertiary hospitals and highest income levels (OR = 1.417, 95% CI 1.121–1.791) were positively associated with assisted ventilation use, whereas non-metropolitan areas (OR = 0.716, 95% CI 0.585–0.875) and medical aid (OR = 0.275, 95% CI 0.163–0.465) revealed a negative relationship.

**Table 2:** Adjusted and unadjusted odds ratio for assisted ventilation in amyotrophic lateral sclerosis.

Variable	Model 1			Model 2			Model 3			Model 4		
	OR	95% CI	*p* value	OR	95% CI	*p* value	OR	95% CI	*p* value	OR	95% CI	*p* value
**Sex**												
Female	1.000			1.000			1.000			1.000		
Male	1.021	0.881–1.183	0.7806	0.954	0.814–1.119	0.5657	0.997	0.846–1.176	0.9734	0.097	0.823–1.152	0.7581
**Age group**												
≤ 49	0.733	0.844–0.931	0.0110	0.807	0.628–1.039	0.0962	0.746	0.574–0.968	0.0267	0.806	0.617–1.053	0.1131
50–59	0.965	0.798–1.168	0.7172	0.985	0.804–1.205	0.8806	1.004	0.814–1.237	0.9722	1.054	0.851–1.304	0.6318
60 ~ 69	1.000			1.000			1.000			1.000		
≥ 70	0.563	0.467–0.680	< .0001	0.491	0.400–0.603	< .0001	0.563	0.456–0.696	< .0001	0.561	0.421–0.698	< .0001
**Nasogastric tube insertion**												
No	1.000			1.000			1.000			1.000		
Yes	2.480	2.080–2.957	< .0001	0.885	0.682–1.130	0.3267	0.849	0.661–1.091	0.2007	0.852	0.659–1.102	0.2223
**Gastrostomy**												
No	1.000			1.000			1.000			1.000		
Yes	2.834	2.325–3.454	< .0001	1.817	1.435–2.301	< .0001	1.472	1.153–1.878	0.0019	1.420	1.107–1.822	0.0058
**Tracheostomy**												
No	1.000			1.000			1.000			1.000		
Yes	9.722	7.153–13.214	< .0001	8.092	5.618–11.656	< .0001	8.514	5.873–12.341	< .0001	9.310	6.357–13.636	< .0001
**Pneumonia**	1.097	1.073–1.122	< .0001	1.073	1.051–1.096	< .0001	1.063	1.041–1.085	< .0001	1.077	1.053–1.102	< .0001
**CCI score**	1.450	1.382–1.522	< .0001				1.385	1.315–1.458	< .0001	1.358	1.288–1.431	< .0001
**Regions**												
Capital	0.995	0.800–1.238	0.9667							0.964	0.753–1.234	0.7723
Metropolitan	1.000									1.000		
Non-metropolitan	0.76	0.636–0.908	0.0025							0.716	0.585–0.875	0.0011
**Income level (quartiles)**												
Lowest	1.000									1.000		
Low-middle	1.492	1.180–1.887	0.0008							1.181	0.897–1.554	0.2355
Middle-high	1.575	1.274–1.948	< .0001							1.269	0.986–1.633	0.0639
Highest	1.624	1.336–1.973	< .0001							1.417	1.121–1.791	0.0036
**Hospital**												
Tertiary Hospital	1.000									1.000		
General Hospital	0.633	0.521–0.770	< .0001							0.583	0.464–0.732	< .0001
Others	0.421	0.319–0.555	< .0001							0.634	0.466–0.863	0.0037
**Type of insurance**												
NHI	1.000									1.000		
Medical aid	0.218	0.141–0.338	< .0001							0.275	0.163–0.465	< .0001

CCI, Charson Comorbidity Index; NHI, National Health Insurance.

Model 1: unadjusted.

Model 2: adjusted for sex, age, nasogastric tube insertion, gastrostomy, tracheostomy, pneumonia.

Model 3: adjusted for sex, age, nasogastric tube insertion, gastrostomy, tracheostomy, pneumonia, CCI score.

Model 4: adjusted for sex, age, nasogastric tube insertion, gastrostomy, tracheostomy, pneumonia, CCI score, region, income level, hospital, type of insurance.

Table 3 shows the OR for both NIV and TIV separately, based on the multivariate logistic regression models. Non-metropolitan area and medical aid were associated with decreased use of assisted ventilation, whereas pneumonia and CCI scores were associated with increased use of assisted ventilation in both NIV and TIV groups. Regarding age groups, patients aged ≥ 70 years showed decreased use of NIV only (OR = 0.535, 95% CI 0.426–0.671). In patients with NIV use, the income level and organization level of hospital revealed a relatively significant and consistent relationship with assisted ventilation use, as compared to those with TIV use. Regarding medical condition, insertion of nasogastric tube showed a negative relationship with NIV use (OR = 0.729, 95% CI 0.161–0.485), whereas a positive relationship was observed with TIV use (OR = 31.12, 95% CI 17.744–49.050).

**Table 3:** Adjusted association for assisted ventilation according to type of ventilation in amyotrophic lateral sclerosis.

	NIV (N = 956)			TIV (N = 272)		
	OR	95% CI	*P* value	OR	95% CI	*P* value
**Sex**						
Female	1.000			1.000		
Male	0.958	0.807–1.138	0.6274	1.311	0.944–1.820	0.1063
**Age group**						
≤ 49	0.082	0.611–1.052	0.1108	0.852	0.460–1.579	0.6116
50–59	1.074	0.865–1.333	0.5199	0.915	0.593–1.412	0.6877
60–69	1.000			1.000		
≥ 70	0.535	0.426–0.671	< .0001	0.785	0.524–1.176	0.2405
**Regions**						
Capital	0.991	0.770–1.275	0.9418	0.725	0.450–1.169	0.1872
Metropolitan	1.000			1.000		
Non-metropolitan	0.727	0.591–0.893	0.0024	0.644	0.436–0.955	0.0285
**Income level (quartiles)**						
Lowest	1.000			1.000		
Low-middle	1.143	0.863–1.513	0.3517	1.129	0.651–1.957	0.6666
Middle-high	1.217	0.940–1.577	0.1363	1.138	0.703–1.842	0.5988
Highest	1.354	1.065–1.721	0.0133	1.258	0.794–1.994	0.3278
**Hospital**						
Tertiary Hospital	1.000			1.000		
General Hospital	0.585	0.461–0.742	< .0001	0.825	0.559–1.216	0.3303
Others	0.639	0.468–0.872	0.0047	0.347	0.153–0.768	0.0112
**Type of insurance**						
National Health Insurance	1.000			1.000		
Medical aid	0.279	0.161–0.485	< .0001	0.308	0.113–0.838	0.0212
**Nasogastric tube insertion**						
No	1.000			1.000		
Yes	0.729	0.565–0.940	0.0149	31.12	19.744–49.050	< .0001
**Gastrostomy**						
No	1.000			1.000		
Yes	1.271	0.978–1.651	0.0730	3.545	2.526–4.977	< .0001
**Pneumonia**	1.087	1.061–1.113	< .0001	1.092	1.061–1.124	< .0001
CCI score*	1.360	1.289–1.434	< .0001	1.309	1.178–1.456	< .0001

Table 4 presents the factors associated with assisted ventilation use according to sex. Based on age, patients aged ≥ 70 years showed decreased use of assisted ventilation in both sexes. According to residential area, only female patients living in non-metropolitan areas showed a significant decreased use of assisted ventilation (OR = 0.594, 95% CI 0.429–0.822). Regarding hospital organization level, a more consistent relationship was observed in females than in males, with more ventilation use in tertiary hospitals than in other hospitals. As for income level, only male patients with the highest income level showed increased assisted ventilation use (OR = 1.427, 95% CI 1.045–1.949).

**Table 4:** Factors associated with assisted ventilation according to sex.

	Male			Female		
	OR	95% CI	*P* value	OR	95% CI	*P* value
**Age group**						
≤ 49	0.850	0.602–1.200	0.3551	0.732	0.479–1.118	0.1448
50–59	0.962	0.733–1.263	0.7881	1.189	0.840–1.683	0.3284
60–69	1.000			1.000		
≥ 70	0.526	0.395–0.702	< .0001	0.601	0.428–0.844	0.0033
**Regions**						
Capital	1.049	0.758–1.451	0.7727	0.821	0.558–1.206	0.3145
Metropolitan	1.000			1.000		
Non-metropolitan	0.792	0.611–1.025	0.0759	0.594	0.429–0.822	0.0017
**Income level (quartiles)**						
Lowest	1.000			1.000		
Low-middle	1.242	0.867–1.778	0.2378	1.121	0.729–1.723	0.6039
Middle-high	1.346	0.971–1.865	0.0748	1.146	0.765–1.715	0.5092
Highest	1.427	1.045–1.949	0.0255	1.414	0.988–2.023	0.0581
**Hospital**						
Tertiary Hospital	1.000			1.000		
General Hospital	0.632	0.467–0.855	0.0030	0.509	0.359–0.722	0.0001
Others	0.682	0.460–1.012	0.0574	0.554	0.337–0.911	0.0201
**Type of insurance**						
National Health Insurance	1.000			1.000		
Medical aid	0.373	0.192–0.722	0.0034	0.172	0.071–0.419	0.0001
**Nasogastric tube insertion**						
No	1.000			1.000		
Yes	0.909	0.643–1.285	0.5904	0.784	0.531–1.156	0.2195
**Gastrostomy**						
No	1.000			1.000		
Yes	1.22	0.868–1.716	0.2522	1.748	1.208–2.528	0.0030
**Tracheostomy**						
No	1.000			1.000		
Yes	10.452	6.263–17.441	< .0001	7.658	4.274–13.723	< .0001
**Pneumonia**	1.079	1.049–1.109	< .0001	1.069	1.026–1.114	0.0013
CCI scores*	1.399	1.304–1.500	< .0001	1.311	1.211–1.420	< .0001

**Figure 2:**
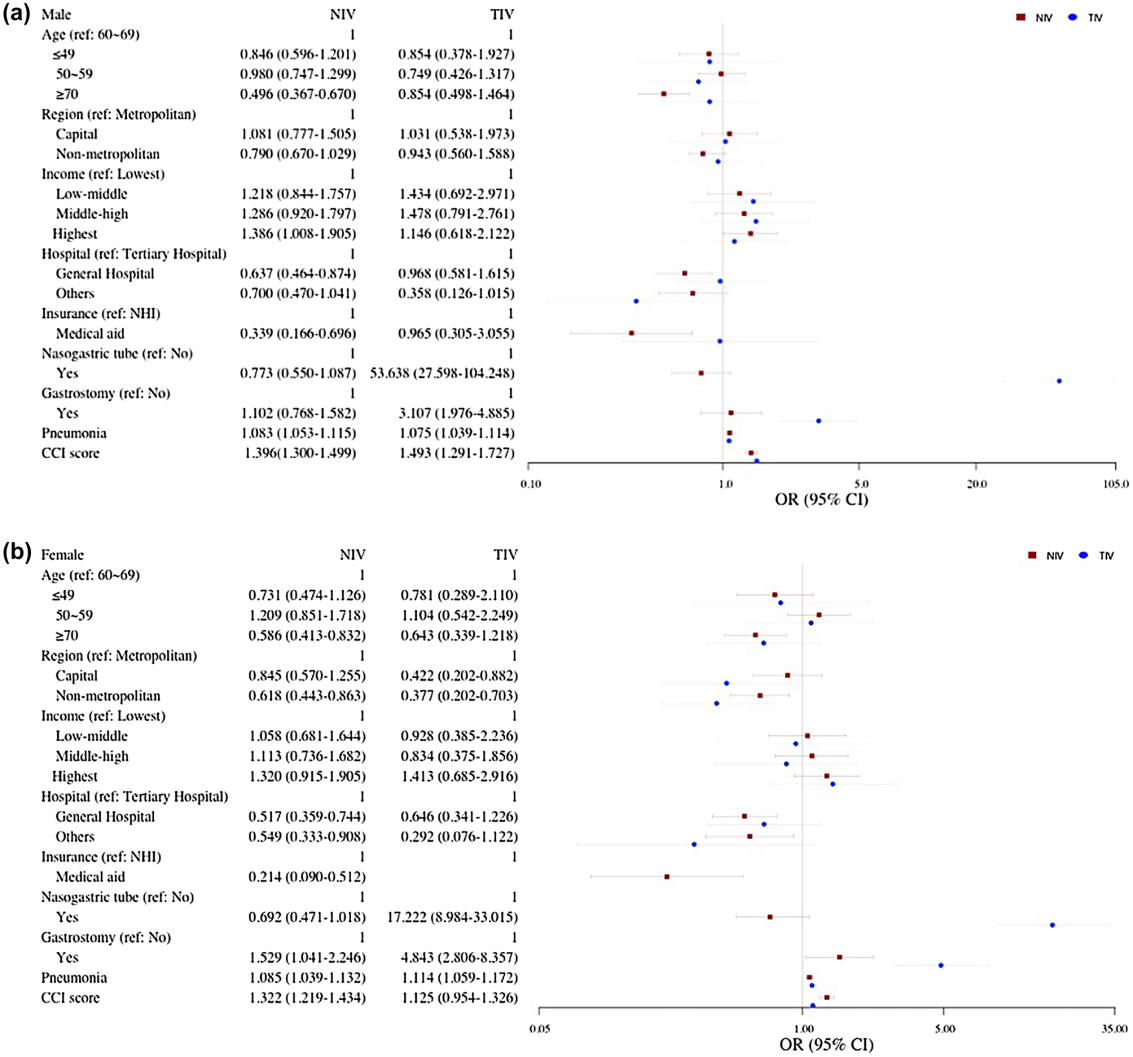
Adjusted odds ratio for assisted ventilation according to type of ventilation in (**a**) males and (**b**) females with amyotrophic lateral sclerosis.

Multivariate regression models for assisted ventilation use according to the type of ventilation, stratified by sex, are shown in Fig. 2. Effects of age, medical condition, and SES on the use of assisted ventilation among patients with ALS remained consistent in subgroup analyses.

## Discussion

In this study, we analyzed 3057 patients with ALS to evaluate the factors associated with assisted ventilation use from a nationwide population-based database comprising the entire Korean population. During the 6-year follow-up period, 1228 (40%) patients with ALS started to use assisted ventilation: 956 patients with NIV and 272 patients with TIV. No significant difference was observed in assisted ventilation use according to sex, and patients aged ≥ 70 years showed decreased use of NIV. Regarding SES, discrepancies for assisted ventilation use in patients with ALS were observed, showing a positive relationship with tertiary hospitals and the highest income level and a negative relationship with non-metropolitan areas and medical aid. When analyzed separately by ventilator type, these discrepancies remained more consistent for NIV use, whereas TIV use was significantly related to medical conditions, including nasogastric tube insertion and gastrostomy.

Although NIV has been recommended as the treatment of choice for respiratory failure in patients with ALS, only a few studies have investigated the factors associated with assisted ventilation use^8,10,12^. Furthermore, ALS is a degenerative motor neuron disease with a low incidence and prevalence, and the number of enrolled patients with ALS in previous studies was relatively small^8,9^. There have been only two longitudinal follow-up studies focusing on the risk factors for assisted ventilation use with more than one thousand participants^10,12^. Thus, we tried to evaluate the risk factors, including SES and medical condition, using a nationwide population-based database covering whole the Korean population.

Our results show no significant difference in assisted ventilation use according to sex in all the models assessed. This result was different from previous studies showing predominant use of assisted ventilation in males compared to females^8,11,12,19^. One possible explanation for this discrepancy of assisted ventilation use in both sexes might be ethnicity. While most studies that showed a male predominance for assisted ventilation use in patients with ALS were performed in Western countries^8,11,12,19^, studies in Asian countries have been limited. ALS is a multifactorial disease resulting from the interaction of genetic and environmental factor^20–22^. The incidence of ALS in the Korean population is relatively low as compared to the European populations^23^, with reports of genetic differences in *C9orf72, TARDBP, and OPTN* genes^13,14^. These genetic differences and cultural factors, such as dietary habits, could affect disease progression, which woold include assisted ventilation use according to sex in the Korean population. Another reason could be sex differences in the utilization of healthcare services. Females have been suggested to have higher utilization rates of healthcare services than males^24,25^. Most previous studies on assisted ventilation use in ALS were conducted in a hospital-based setting or from the data of the ALS registry for research^8,10,11^. This was a nationwide claims data-based study, thus, high utilization of medical facilities could lead to increased use of assisted ventilation in females. Although there was no difference in assisted ventilation use according to sex in our results, we found that different patterns of discrepancies existed for the use of assisted ventilation by sex. Only female patients living in non-metropolitan areas showed decreased use of assisted ventilation, whereas high income levels showed a positive relationship with assisted ventilation use only in males. Since previous reports of risk factors for assisted ventilation use stratified by sex are scarce, future studies on this matter would be helpful determining the reasons for male predominance on assisted ventilation use in previous studies.

Regarding age groups, age ≥ 70 years was found to be a barrier to assisted ventilation use, especially NIV use. Effects of age on assisted ventilation use have been controversial in previous studies. Although some studies have suggested older^9^ or young age^10^ at diagnosis as a risk factor for assisted ventilation use, others have reported no relationship between age and assisted ventilation use^8^. Various factors, such as life expectancy increase, cultural factors and health care policy, might have influenced the different results across studies. A previous population-based study in Korea suggested that survival time from ALS onset to death was significantly decreased as age increased^23^, and our results suggest that this may be related to low use of assisted ventilation in the elderly. There might be a tendency towards lesser use of assisted ventilation in patients aged ≥ 70 years due to low life expectancy in real-world practice. It has been previously reported that attitudes of physicians toward TIV can strongly affect patient decisions^26^, which might be similar to the results for NIV use observed in this study. In the management of ALS, religious factors, cultural factors, and personal decision have been suggested as important factors^27,28^. There have even been studies reporting the influence of spirituality and religion on quality of life and decision-making in interventions related to death^28,29^. Since direct relationship between religious factor and ventilator use has been rarely studied, future studies with consideration of religious factors and personal decision are warranted.

Attitudes favoring any medical treatment have been suggested to be related to the increased use of assisted ventilation^8,12^. Gastrostomy and use of speech valves have been associated with assisted ventilation use in previous studies^8,19^. Similarly, gastrostomy was also related with increased use of TIV in our results. Nasogastric tube insertion was found to significantly lower the use of NIV but increase the use of TIV. The use of interfaces, such as masks or nasal prongs, for NIV might be intolerable for patients with ALS with a nasogastric tube, thus increasing the use of TIV tremendously (OR = 31.12, 95% CI 19.744–49.050). On the other hand, medical conditions, including recurrent pneumonia, and high CCI score also showed a positive relationship with both NIV and TIV use. Pneumonia has been suggested as a major cause of death in ALS^30,31^, and it has been reported to be more frequent in patients with NIV^32^. The effects of recurrent pneumonia on assisted ventilation use have been scarcely investigated, and our results indicate that pneumonia was significantly associated with increased use of assisted ventilation use. Medical conditions, including comorbidities and recurrent pneumonia, require more attention in terms of management strategies in patients with ALS.

ALS imposes considerable socioeconomic burden^16,33^. Although many countries operate on a public healthcare system, there have been reports on personal economic impact of ALS, as measured by non-reimbursed, out-of-pocket expenses^17,33^. In our results, disparities in assisted ventilation use was observed among patients with ALS. High income levels^8,19^ and tertiary ALS centers^10^ were associated with increased use of assisted ventilation. The type of insurance was not related to assisted ventilation use in previous studies, which is contrary to our results that showed reduced use of assisted ventilation in the medical aid group^8,19^. Given that healthcare management systems differ across countries, this would also affect the use of assisted ventilation differently. For example, we categorized the type of insurance into two groups in this study, whereas this factor was categorized into five groups in a previous study in the USA^8^. When analyzed separately by ventilator type, income level was only associated with NIV use, additionally reporting a decreased use of assisted ventilation in general hospitals than in tertiary hospitals. NIV use was more influenced by SES than TIV, whereas TIV use showed a significant relationship with medical conditions such as gastrostomy and nasogastric tube insertion.

Proposed criteria for NIV on the European guidelines for the clinical management of ALS consists of symptoms or signs related to respiratory muscle weakness and abnormal respiratory function tests^34^. The utilization of assisted ventilation varies across countries. NIV use was 23% in Australia^35^, 21% in Germany^36^, 34% in United States^12^, 27% in Japan^37^, and 17% in Taiwan^38^. TIV also showed various utilization rate: 9.5% in Germany^36^, approximately 11% in Italy^39^, above 20% in Japan^37^, and 21% in Taiwan^38^. These differences in the utilization rates of assisted ventilation could be attributed to various factors, including genetics, healthcare policies covering ventilator use, cultural factors (e.g., religion), and study design such as follow up duration or type of ALS involved in the study. Regarding genetic differences, C9orf72 repeat expansion has been identified as the causative mutation in ALS^40^. High frequencies of repeat expansion, ranging from 25 to 50% in familiar ALS cases and 4–7% in sporadic ALS cases, have been reported in Italy, Germany, Belgium, UK, and the United States^40,41^. In contrast, the C9orf72 repeat expansion was not a major contributor in Korean patients with ALS, as in other Asian countries^42–44^. The C9orf72 expansion mutation has been reported to be related to impairment of behavioral and cognitive function^45^, indicating that the relatively preserved cognitive function of ALS patients in Korea could have influenced assisted ventilation use. In our results, the utilization of NIV was 31% and that of TIV was 9%, which were not similar with those in Asian countries, possibly attributing to various factors in each country. Thus, future studies to prevent underutilization of assist ventilation with the consideration of cultural and individual factors are warranted for optimal management of ALS.

### Limitations

Despite the study findings, several limitations should be noted. First, we could not include respiratory symptoms, disease severity, disease type (bulbar or spinal), and results of pulmonary function test in the analyses. Since this study used a nationwide claims-based database, these data were unavailable. In order to partially represent disease progression, we included medical conditions such as nasogastric tube insertion, gastrostomy, and tracheostomy. Second, we only used residential area, insurance types, income level and organization level of hospital to represent SES. Additional information, such as education level and marital status, could have provided more profound interpretations for the use of assisted ventilation in patients with ALS. Third, in this claims-based study, individual factors regarding religion or personal decision were difficult to obtain, which might have affected the utilization of assisted ventilation. Lastly, survival analysis was not performed in this study, only the mean survival times for NIV and TIV groups were reported. The follow-up period for ALS patients in this study ranged from one to six years, which was somewhat short to perform survival analysis. The similar mean survival times between NIV and TIV groups in our results might originate from this relatively short follow-up period. Future studies of assisted ventilator-related survival in ALS with longer follow-up duration are warranted.

## Methods

### Data source and study populations

Korea has maintained a nationwide health insurance system since 1963 under the Korean National Health Insurance Service, in which nearly all the data in the health system have been centralized. This service is a single-payer system that provides mandatory universal comprehensive medical coverage of over 97% of the Korean citizens and medical aid to approximately 3% of the population in the lowest income level. The gathered data includes a unique anonymous number for each patient and summarizes demographics such as age, sex, type of insurance, a list of diagnoses according to the International Classification of Diseases (ICD-10), healthcare utilization and claimed medical costs.

The Korean government has started to operate a registration program for rare intractable diseases (RID), including ALS, to assist patients with their medical expenses in 2001. Since diagnosis is based on strict diagnostic criteria provided by the RID system in charge of the reimbursement of expenses, the RID data are considered very reliable. For the registration of an ALS diagnosis, both clinical examination findings of upper and lower motor neuron signs and the verification of neurogenic changes through electromyography are required.

We selected patients with primary or secondary diagnoses of ALS (ICD-10 code: G12.21) and a registration code for ALS (V123) in the registration program for RID between 2011 and 2017. We then excluded individuals who were diagnosed with ALS in 2011 to ensure that only those with new ALS episodes were enrolled. Patients who had used assisted ventilation before the diagnosis of ALS were excluded. This study was approved by the Institutional Review Board of the Seoul National University Hospital, which waived the need for informed consent (E-2009–108-1158). This study was performed in accordance with the Declaration of Helsinki.

### Outcome: assisted ventilation

In this study, we evaluated factors associated with assisted ventilation, including both NIV and TIV. In Korea, for the application of assisted ventilation, patients had to satisfy both the following inclusion criteria: (1) more than two symptoms of hypercapnia, and (2) two episodes of hypercapnia (PaCO_2_ ≥ 45 mmHg on arterial blood gas analysis or PaCO_2_ ≥ 40 on end-tidal respiration) on different days. To define TIV, we used procedure codes (O1300, O1301, O1303, O1305, or O1306) for tracheostomy combined with assisted ventilation. We only included assisted ventilation after the diagnosis of ALS to identify the factors associated with assisted ventilation use in patients with ALS.

### Other variables

Demographic characteristics, including age, sex, residential area, income level, organization level of hospitals, and type of insurance, were analyzed. Ages were categorized into four groups: ≤ 49 years, 50–59 years, 60–69 years, and ≥ 70 years. Residential areas were categorized into capital, metropolitan and non-metropolitan areas. In Korea, there are six metropolitan cities with populations exceeding one million people: Busan, Incheon, Daejeon, Daegu, Gwangju and Ulsan. Patients living in these cities were categorized as living in a ‘metropolitan’ area. Patients living in areas other than Seoul, the capital of Korea, and the metropolitan cities mentioned above were classified as living in ‘non-metropolitan’ areas. For income level, we used the average monthly insurance premium for household income as a proxy variable. People who have National Health Insurance (NHI) based on employment pay a monthly insurance premium according to annual salary, and people who are self-employed pay for their premium based on the value of their property. The income deciles of enrolled participants were categorized into four categories: Q for all medical aid enrollees + 0–25 percentiles of NHI enrollees, Q2 for 26–50 percentile of NHI enrollees, Q3 for 51–75 percentile of NHI enrollees, and Q4 for 76–100 percentile of NHI enrollees. Organization level of hospitals was categorized into three groups: tertiary general hospitals, general hospitals, and others. Comorbidity was defined using the Charlson Comorbidity Index (CCI). The diseases included in the CCI include congestive heart failure, myocardial infarction, cerebrovascular disease, peripheral vascular disease, connective tissue disease, chronic lung disease, ulcer, chronic liver disease, severe liver disease, dementia, diabetes, hemiplegia, moderate or severe kidney disease, tumor, leukemia, lymphoma, moderate or metastatic solid tumor and acquired immunodeficiency syndrome. We also included factors that are important in ALS progression, such as pneumonia, gastrostomy and nasogastric tube insertion, in the analyses.

### Statistical analyses

Patients were divided into two groups to investigate the risk factors for assisted ventilation use: patients with assisted ventilation and those without assisted ventilation. Variables in the two groups were compared using Student’s t-test or the chi-square test. We then estimated the adjusted OR and 95% CI for the relationships between the covariates and assisted ventilation use by applying univariate and multivariate logistic regression models. Regression modelling was performed using the SAS version 9.4 (SAS Institute, Cary, NC, USA) software, and a *P* < 0.05 was considered statistically significant for all analyses.

### Research involving human participants and/or animals

This article does not contain any studies with human participants or animals performed by any of the authors.

### Ethical approval

This study was approved by the Institutional Review Board of Seoul National University Hospital, which waived the need for informed consent (E-2009–108-1158).

### Informed consent

The nature of this article did not require informed consent.

## Abbreviations

ALS: Amyotrophic lateral sclerosis
CCI: Charlson Comorbidity Index
CI: Confidence interval
FVC: Forced vital capacity
NHI: National Health Insurance
NIV: Non-invasive ventilation
OR: Odds ratio
RID: Rare intractable diseases
SES: Socioeconomic status
TIV: Tracheostomy invasive ventilation
